# Africa's health: could the private sector accelerate the progress towards health MDGs?

**DOI:** 10.1186/1755-7682-4-39

**Published:** 2011-11-25

**Authors:** Luis G Sambo, Joses M Kirigia

**Affiliations:** 1 World Health Organization, Regional Office for Africa, P.O. 6, Brazzaville, Republic of Congo

## Abstract

**Background:**

Out of 1.484 billion disability-adjusted life years lost globally in 2008, 369.1 million (25%) were lost in the WHO African Region. Despite the heavy disease burden, the majority of countries in the Region are not on track to achieve Millennium Development Goals (MDG) 4 (reducing child mortality), 5 (improving maternal health), and 6 (combating HIV/AIDS, malaria and other diseases). This article provides an overview of the state of public health, summarizes 2010-2015 WHO priorities, and explores the role that private sector could play to accelerate efforts towards health MDGs in the African Region.

**Discussion:**

Of the 752 total resolutions adopted by the WHO Regional Committee for Africa (RC) between years 1951 and 2010, 45 mention the role of the private sector. We argue that despite the rather limited role implied in RC resolutions, the private sector has a pivotal role in supporting the achievement of health MDGs, and articulating efforts with 2010-2015 priorities for WHO in the African Region: provision of normative and policy guidance as well as strengthening partnerships and harmonization; supporting the strengthening of health systems based on the Primary Health Care approach; putting the health of mothers and children first; accelerating actions on HIV/AIDS, malaria and tuberculosis; intensifying the prevention and control of communicable and noncommunicable diseases; and accelerating response to the determinants of health.

**Conclusion:**

The very high maternal and children mortality, very high burden of communicable and non-communicable diseases, health systems challenges, and inter-sectoral issues related to key determinants of health are too heavy for the public sector to address alone. Therefore, there is clear need for the private sector, given its breadth, scope and size, to play a more significant role in supporting governments, communities and partners to develop and implement national health policies and strategic plans; strengthen health systems capacities; and implement roadmaps for accelerating the attainment of health MDGs relating to maternal and child health, reducing disease burden, and promoting social determinants of health.

In order for governments to further explore the potential benefits of the private sector towards improved performance of health systems, there is need for accurate evidence on the private sector capacity in areas of prevention, promotion, treatment and rehabilitation; dialogue and negotiation; clear definition of roles and responsibilities; and regulatory frameworks.

## Background

The objectives of this article are to provide an overview of the state of public health in the 46 countries of the African Region; summarize 2010-2015 priorities for WHO in the African Region; analyze the role of the private sector as perceived by the WHO Regional Committee for Africa; and provide the author's view points on the potential role of the private sector in health development. The private sector refers to the part of the economy not under direct state control. In this article, the working definition of private sector is any private enterprise, business or businesses not under state control, i.e. run by private individuals or groups for profit.

### Overview of the state of public health

**Figure 1:**
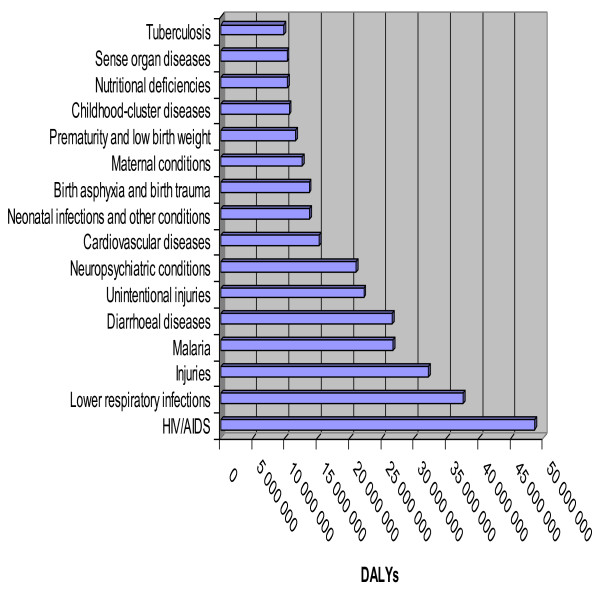
**Top 16 causes of disability adjusted life years (DALYs) loss in WHO African Region, 2008**.

In 2008, the world lost 1.484 billion disability-adjusted life years (DALYs) from various causes. The WHO African Region bore 25% of that burden, while sharing just 12.1% of the world's population [1]. Of the total DALYs lost in the African Region, 83% resulted from the 16 causes depicted in Figure 1. HIV/AIDS, lower respiratory infections, injuries, malaria and diarrhoeal diseases accounted for 47% of the DALYs lost in the Region.

In 2000 the world leaders agreed on eight Millennium Development Goals (MDGs) to address disease, income poverty, hunger, ignorance, and squalor [2]. The MDGs 4 and 5 are about health of children and women, and MDG6 is concerned with control main diseases such as HIV/AIDS, tuberculosis and malaria. Progress towards the MDG targets has been uneven in Africa [3].

African Region under-five mortality rate decreased from 179 per 1000 live births in 1990 to 127 in 2009 [4]. However, it is declining at an average rate of 1.45% per year, which is slower than the 8% per year needed to achieve MDG4 by 2015. The key to progress towards attaining this goal by 2015 is to reach every newborn and child with priority interventions such as breast feeding; immunization to curb vaccine-preventable diseases; and prevention and management of common childhood illnesses such as pneumonia, acute respiratory infections, diarrhoea, malaria, malnutrition and HIV infection. For instance, immunization coverage among one-year-old children for the third dose of DPT3 increased in the African Region from 57% in 1990 to 71% in 2009 [4].

Maternal mortality ratio also decreased from 850 per 100 000 live births in 1990 to 620 in 2008 [4,5]. Most countries are unlikely to attain the MDG 5A target at the prevailing low rate of decline in maternal mortality ratio. Majority of maternal deaths could be averted if every woman in need had access to quality reproductive health services such as skilled attendance during pregnancy, childbirth and the postnatal period; and emergency obstetric care (EOC) and family planning. Unfortunately, coverage of skilled birth attendance in the African Region remains low at 48%, with wide variation in the rates among countries [4]. Only 44% of pregnant women made at least four antenatal care visits in 2010 [4] and even a smaller proportion of pregnant women in need of EOC got it.

The world leaders committed to halt and reverse the incidence of HIV/AIDS, Malaria and TB by 2015 as part of efforts to achieve MDG6. Yet, whereas the African Region is home to approximately 12% of the world population, it accounts for two thirds (67.6%) of people living with HIV/AIDS globally; two thirds (68.2%) of all new adult HIV infections; 90% of new HIV infections in children; and 72.2% of AIDS-related deaths [6]. The number of AIDS-related deaths decreased from 1, 400, 000 in 2001 to 1, 300, 000 in 2009. The number of new HIV infections per year is on decline on average, but in general the incidence rate is till very high. Coverage of primary prevention interventions is low. For example, in 2009 only 33% of Male and 25% of female population aged 15-24 years had comprehensive correct knowledge of HIV/AIDS [4]. The prevalence of condom use by adults aged 15-49 years during higher risk sex is also low. Coverage of services for the prevention of mother-to-child transmission was 54% in 2009 [4]. Though Africa has made significant progress in increasing access to antiretroviral treatment, there is still large unmet need.

The African Region also accounts for about a third (27.9%) of all tuberculosis (TB) cases. The prevalence rate of TB increased from 450 per 100 000 population in 2000 to 475 per 100 000 population in 2009; and incidence of TB increased from 314 per 100 000 population in 2000 to 345 per 100 000 population per year [4]. The situation has been exacerbated by the combination of HIV with TB and increase in prevalence of MDR-TB. Malaria in Africa represents 85% of all malaria cases; and 89% of all malaria-related deaths worldwide [7]. In some countries the battle against malaria is progressing with growing coverage of vector control using insecticide-treated nets and indoor residual spraying; artimisinin-based combination therapy; and intermittent preventive treatment of malaria in pregnancy.

### Strategic thrusts for WHO in the African Region

Based on the above overview of the state of public health in the Region, a number of thrusts were established for WHO in the African Region from 2010 to 2015 [8].

#### Providing normative and policy guidance as well as strengthening partnerships and harmonization

This entails providing technical support to governments to develop comprehensive (including the private health sector) national health policies and costed national health sector strategic plans [9]. It also includes implementing and monitoring the Paris Declaration on Aid Effectiveness [10] and the Accra Agenda for Action [11].

#### Supporting the strengthening of health systems based on the Primary Health Care approach

This involves supporting countries to implement the Ouagadougou Declaration [12] on Primary Health Care and health systems strengthening and the Algiers Declaration [13] on Research for Health in Africa. The overall objective is to strengthen leadership and governance in health; health workforce capacity [14]; adequate provision and use of medical products, vaccines and technologies [15,16]; health financing with a view to strengthening the delivery of health care; and improving health outcomes [17]. It also involves implementing the 'Health financing: a strategy of the WHO African Region' [18]; and the Seychelles [19] and Cape Verde [20] Declarations of Ministers of Health of the Small Island Developing States in the African Region.

#### Putting the health of mothers and children first

This requires supporting countries to implement the Roadmap for accelerating the attainment of the MDGs relating to maternal and newborn health in Africa [21]. It includes defining a minimum package of services at each level of the health system; assessing needs; and producing a skilled health workforce for delivery of quality maternal and child health care. This strategic direction is also about accelerating the implementation of the Child Survival Strategy for the African Region [22].

#### Accelerating actions on HIV/AIDS, malaria, tuberculosis and other communicable diseases

This provides accelerated support to countries to expand coverage of cost-effective interventions into HIV/AIDS [23-29], tuberculosis [30-32] and malaria [33-37]. It also includes support to address the burden of neglected tropical diseases, e.g. dracunculiasis [38], leprosy [39], lymphatic filariasis, onchocerciasis [40], trypanosomiasis [41], schistosomiasis, soil-transmitted helminths and trachoma); and to improve systems of early response to epidemics and pandemics [42]. In addition, countries are supported to comply with the core requirements of the International Health Regulations [43].

#### Intensifying the prevention and control of noncommunicable diseases

This is about intensifying support to countries to prevent and control the increasing burden of noncommunicable diseases [44] such as cancer [45], diabetes [46], and cardiovascular diseases, as well as conditions related to oral health [47], mental health and substance abuse [48]. It also entails implementation of the regional strategy on Integrated Disease Surveillance and Response (IDSR), which aims to improve the availability and use of surveillance and laboratory data for control of priority infectious diseases [42]; and policy orientations on the establishment of centres of excellence for disease surveillance, public health laboratories, food and medicines regulation [49].

#### Accelerating response to the determinants of health

This concerns implementing the Libreville declaration on health and environment [50], the Luanda commitment [51] by African Ministers responsible for health and environment to implement the Libreville declaration, the Nairobi call for action for closing the implementation gap in health promotion [52], and the regional strategies on environmental health [53], food safety [54], health promotion [55], poverty and health [56], and addressing the key determinants of health in the African Region [57]. Implementation includes addressing risk factors such as unhealthy food consumption patterns, harmful use of alcohol, tobacco use, sedentary lifestyle, and other behavioural challenges.

The prevailing situation can only be changed if governments, communities, the private sector and development partners work together to accelerate achievement of the Millennium Development Goals.

## Discussion

### Analysis of Ministers of Health deliberations on the role of private sector in health development

**Figure 2:**
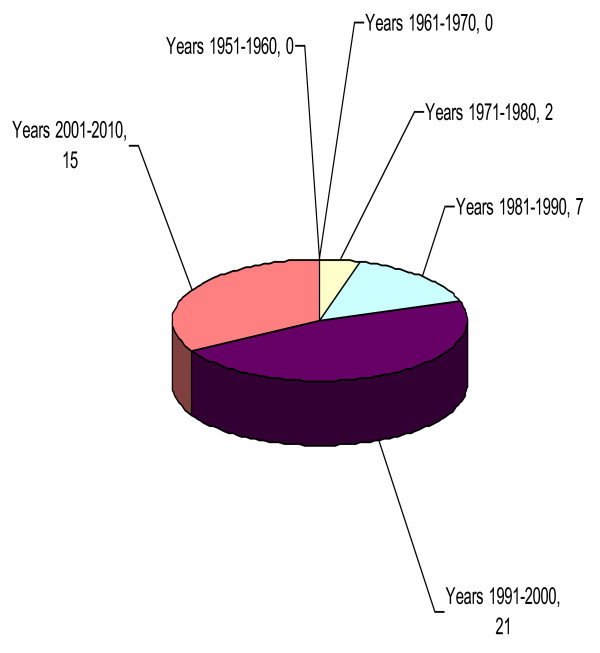
**Number of Regional Committee resolutions mentioning the private sector, 1951-2010**.

Since the WHO Regional Office for Africa was established in 1951, the WHO Regional Committee for Africa has held 60 sessions and adopted 752 resolutions [58-63]. The number of resolutions increased between 1951 and 1970 but decreased in a sustained manner since 1971. Of the total resolutions adopted between 1951 and 2010, only 45 (6%) mention the private sector. Figure 2 shows that during the first 25 years of the Regional Committee, there was no mention of the private sector in any of the 370 resolutions adopted.

The role of the private sector was first mentioned during the Regional Committee session in 1976. Over the following 15 years, from 1976 to 1990, the private sector was mentioned in nine of the 212 resolutions adopted. From 1991 to 2005, the role of the private sector was considered in 31 of the 141 resolutions adopted. During the last five years, from 2006 to 2010, the private sector features in five of the 29 resolutions adopted. Since 1976, there was a steady increase in the number of resolutions acknowledging the role of the private sector in health development in the Region reaching a peak in the decade 1990-2000. In 2001-2010, the number of resolutions concerning the private sector decreased slightly, mainly due to a decreasing trend in the total number of resolutions adopted by the Committee.

Table 1 shows the areas in which the private sector has been expected to play a role. Of the 45 resolutions involving the private sector role in public health, 11 (24%) were on prevention and control of HIV/AIDS, tuberculosis and malaria; nine (20%) were on communicable diseases; two (4%) were on noncommunicable diseases; five (11%) were on family and reproductive health; eight (18%) were on management of health risk factors; and ten (22%) were on health systems strengthening. The three areas with the highest number of resolutions underscoring the private sector role were HIV/AIDS, dracunculiasis and health technologies (essential medicines, vaccines and equipment).

**Table 1:** Broad and specific areas where private sector is expected to play a role

*Broad thematic areas*	*Specific areas*	*Number of Regional Committee resolutions*
HIV/AID, tuberculosis and malaria	HIV/AIDS	5
	Tuberculosis	4
	Malaria	2
Prevention and control of other communicable diseases	Trypanosomiasis	1
	Dracunculiasis	5
	Onchocerciasis	2
	Cholera	1
Non-communicable disease prevention and control	Cardiovascular diseases	1
	Diabetes	1
Family and reproductive health	Child health	2
	EPI	1
	Family planning	1
	Maternal and newborn health	1
Health risk factors management	Food safety	1
	Nutrition	1
	Iodine deficiency disorders	1
	Health and development	2
	Environmental sanitation	1
	Tobacco	1
	Health promotion	1
Health systems strengthening	Essential medicines, vaccines and technologies	5
	E-Health	1
	Information systems	1
	Workforce	1
	Health services	1
	Health sector reforms	1

The question remains as to why only 6% of the Regional Committee resolutions pertain, either directly or indirectly, to the role of the private sector in health development. One answer may be that most of the resolutions address public health problems whereas the private sector seems more interested in clinical services. Secondly, there may be limited awareness of the private sector's potential scope and role in health development. A third answer could be that health economics, a discipline that could have raised awareness of the private sector role in health development, only emerged in the mid-1970s. A fourth reply could be that the private sector has not made an organized effort to demonstrate its potential capacity to contribute to the public good. These speculations are presented here for analysis and better understanding of the current situation.

### Potential role of the private sector in expanding healthcare services

This section provides author's opinions on the potential role of the private sector in supporting implementation of the thrusts/strategies mentioned earlier.

#### Thrust 1: Providing normative and policy guidance as well as strengthening partnerships and harmonization

National health policies and national health strategic plans ought to encompass the public, nongovernmental organization and private health sectors. At both development and revision stages, private service providers, through their associations, can work with national authorities so that their contributions are mainstreamed into these and other health sector strategic documents. In relation to this process, the private sector can provide various data for National Health Management Information Systems. Such information should include inputs (expenditures on pharmaceutical and non-pharmaceutical supplies, number of health facilities, beds, clinical technologies, and health workers); outputs (number of inpatient admissions and discharges, number of preventive and curative outpatient visits); and outcomes. The private sector could also facilitate implementation of national public health legislation and the International Health Regulations [43].

#### Thrust 3: Supporting health systems strengthening based on the Primary Health Care approach

The private sector has a contribution to make in strengthening major components of national health systems, i.e. delivery of health services; health workforce; health information; medical products, vaccines and technologies; and health financing [17].

##### Delivery of health services

In any health system, good health services are those which deliver effective, safe, timely and quality personal and non-personal care to those that need it with minimum waste [17]. In terms of provision of health services, a government may leverage the private sector to construct and maintain health facilities; supply and maintain equipment; provide utilities such as power, water and waste management services to public facilities; provide catering, cleaning, laundry and transport services to health facilities; and provide health care to populations in remote areas. Governments can also create an enabling policy and regulatory environment for the business community to invest in private sector owned and managed health care enterprises, e.g. private hospitals, clinics, and community-based health promotion services.

##### Health workforce

A country's health workforce consists broadly of health service providers and health management and support workers. Most countries of the African Region, especially the 36 that are in a health workforce crisis [14], may require private sector support in order to produce an adequate health workforce. This may entail private sector partnering with public health schools, medical schools, and schools of nursing. Such public-private partnerships may mean that private education institutions offer health training courses through the public education system; receive public funds to produce agreed quantities of human resources for health; provide capital to individuals or groups to establish accredited private universities and colleges; and pay public health training institutions to produce health workforce for them or even for continuing education.

##### Health information

A well-functioning health information system is one that ensures the production, analysis, dissemination and use of reliable and timely information on health determinants, health systems performance and health status [17]. The appreciation of the role of the private sector partly hinges on the quality of information submitted to the National Health Information System (NHIS). However, the private sector in most countries does not regularly provide data to the NHIS, and thus, it is very difficult for ministries of health to generate evidence for comprehensive health sector plans. Therefore, in terms of health information, there is room for the private sector to provide statistical information; make relevant investments in eHealth infrastructure and services; and fund or conduct biomedical and health systems research aimed at discovery or improvement of health technologies and improvements in access to quality health care.

##### Medical products, vaccines and technologies

A well-functioning health system ensures equitable access to essential medical products, vaccines and technologies of assured quality, safety, efficacy and cost effectiveness, and their scientifically sound and cost-effective use [17]. The private sector can complement government efforts in improving health logistics in specific aspects such as procurement and storage of medical products, vaccines and other technologies as well as collaborate in the enforcement of drug regulatory mechanisms and pharmacovigilance. The latter entails collecting, monitoring, researching, assessing and evaluating information from healthcare providers and patients on the adverse effects of medications, biological products, blood products, vaccines, medical devices, herbal medicines, and complementary and traditional medicines with a view to identifying new information about hazards associated with medicines and preventing harm to patients [64]. It is important to emphasize that national governments have a fundamental role in oversight and regulatory matters.

##### Health financing

A good health financing system raises adequate funds for health to ensure that people who needed health services are protected from any financial catastrophe associated with having to pay for them [17]. Almost 50% of total health expenditures in the African Region are from private spending; of which, approximately 71% of expenditures are from direct household out-of-pocket payments to various health service providers. These direct payments are not pooled to share financial risk, and thus, may expose a large proportion of the population to financial catastrophe and impoverishment [65]. On matters of health financing, the private sector can contribute in strengthening national health financing strategies by assessing the economic feasibility of prepaid health schemes, e.g. health insurance; developing or expanding coverage of private health insurance; and paying premium contributions to national social health insurance schemes for employees and families.

#### Thrust 3: Putting the health of mothers and children first

With regard to reducing maternal and newborn morbidity and mortality, the private sector can play a role by producing skilled birth attendants; increasing the number of private health facilities, especially in rural areas; ensuring that private health facilities provide emergency obstetric care and newborn plus child health services; operating transport businesses, including ambulance services, especially to refer patients and reduce delays in accessing care; establishing micro-financing income-generating schemes, especially in rural areas and mainly for women to improve their financial capacity; and broadcasting Ministry of Health messages geared at modifying deep-seated cultural practices that are detrimental to women's health, e.g. female genital mutilation, delivering without assistance as an indicator of womanhood.

With regard to reductions in child morbidity and mortality, the private health sector can ensure that facilities provide at least a minimum package of interventions. These may include routine immunization; insecticide-treated nets for pregnant women and infants; antenatal care; neonatal care; complementary infant feeding; oral rehydration therapy; malaria treatment; management of pneumonia in newborns and children; antiretroviral drugs for the management of paediatric AIDS; and family planning services [22].

#### Thrusts 4 and 5: Accelerated actions on HIV/AIDS, malaria and tuberculosis; and prevention and control of other communicable and noncommunicable diseases

Concerning public health interventions for reducing the burden of disease, the private sector can play an increasing role in providing support to governments. Private enterprise can produce and supply at affordable prices quality-assured health technologies including laboratory diagnostic tests; first-line and second-line medicines [66,67]; and preventive supplies such as vaccines, condoms, intrauterine contraceptive devices, and long-lasting insecticide-treated nets.

To assist in the prevention and control of noncommunicable diseases, the private sector can assist in implementing the relevant articles of the WHO Framework Convention on Tobacco Control [68]. The private sector can promote physical activity by creating recreational space when constructing commercial and residential projects; providing pedestrian and cyclist pavements when constructing roads; and investing in sports, recreation and leisure facilities, among others. Food industries can promote healthy diets by reducing salt levels, eliminating trans-fatty acids, decreasing saturated fats, limiting free sugars [69] and providing accurate nutrition facts. The private sector can contribute to the reduction of harmful use of alcohol by avoiding sales to under-age consumers; providing information on risks associated with alcohol abuse; barring their employees from driving or operating machinery while under the influence of alcohol; and providing treatment for employees with alcohol-use disorders. Finally, the private sector can help perform environmental assessments of major construction projects (e.g. dams, bridges, airports, ports, oil drilling, irrigation schemes, etc) to ensure that negative public health implications are duly identified and mitigated in a timely manner.

#### Thrust 6: Accelerating response to the determinants of health

There is ample evidence that health is significantly influenced by the socioeconomic conditions in which people live, work and age, as well as the existing healthcare systems [57,70]. According to the WHO Commission on Social Determinants of Health, these socioeconomic conditions can help create or destroy human health [70]. The private sector can contribute to improving the social determinants of health through income and wealth generation and distribution, early childhood care, education, working conditions, job security, food security, gender, housing, safe water and sanitation, and social safety nets. These will play a vital role in improving the health status of people. Therefore, addressing health risks and determinants requires active participation from all sectors.

## Conclusion

The provision of health care within functional/well performing health systems; and addressing challenges related to broad determinants of health in the African Region is too heavy for the public sector to tackle alone. Given its breadth, scope and size, the private sector, therefore, should play a more significant role in supporting government efforts to develop and implement national health policies and strategic plans; strengthen the national health systems; reduce maternal, newborn and child morbidity and mortality; reduce the burden of disease, i.e. communicable, noncommunicable and neglected tropical diseases; and improve the broad determinants of health.

All of the above should be done bearing in mind the limited purchasing capacity of a significant proportion of people in Africa. Merely increasing the supply of health services is not sufficient for health development. Governments and the private sector, among other health stakeholders, will need to collaboratively manage the quality, cost and access to health care and other key determinants of health in order to improve health status, quality of life and human development.

In conclusion, it is relevant to reiterate some of Harding's discussion about private participation in health systems [71]. He identified three factors necessary for harnessing the private sector to increase its role and remain an effective player in health systems: knowledge (on the part of policy-makers) about the private sector; ongoing dialogue between public and private stakeholders; and institutionalized policy instruments (especially financing, regulation, and information) for interacting with the private sector. To fulfil these prerequisites, governments need to build capacities for planning, negotiation, oversight and regulation [72,73], including those for regulation of prices, services, entitlements, quality of care, health facility licensing, health worker accreditation, medical audits, research, and clinical protocols, to mention a few. In addition to legislation and other controls, governments can influence the private sector through incentives and market structuring [71].

## Competing interests

The authors declare that they have no competing interests.

## Authors' contributions

LGS and JMK contributed to the design, literature review, analysis and writing of various sections of the manuscript. Both authors read and approved the final manuscript.
